# GeneCloudOmics: A Data Analytic Cloud Platform for High-Throughput Gene Expression Analysis

**DOI:** 10.3389/fbinf.2021.693836

**Published:** 2021-11-25

**Authors:** Mohamed Helmy, Rahul Agrawal, Javed Ali, Mohamed Soudy, Thuy Tien Bui, Kumar Selvarajoo

**Affiliations:** ^1^ Bioinformatics Institute (BII), Agency for Science, Technology and Research (A*STAR), Singapore, Singapore; ^2^ Department of Computer Science, Lakehead University, Thunder Bay, ON, Canada; ^3^ Department of Geology and Geophysics, Indian Institute of Technology (IIT) Kharagpur, Kharagpur, India; ^4^ Proteomics and Metabolomics Unit, Children Cancer Hospital (CCHE-57357), Cairo, Egypt; ^5^ Singapore Institute of Food and Biotechnology Innovation (SIFBI), Agency for Science, Technology and Research (A*STAR), Singapore, Singapore; ^6^ Synthetic Biology for Clinical and Technological Innovation (SynCTI), National University of Singapore (NUS), Singapore, Singapore

**Keywords:** OMICS data, gene expression analysis, bioinformatics, microarray, RNA-seq, transcriptomics, data analytics

## Abstract

Gene expression profiling techniques, such as DNA microarray and RNA-Sequencing, have provided significant impact on our understanding of biological systems. They contribute to almost all aspects of biomedical research, including studying developmental biology, host-parasite relationships, disease progression and drug effects. However, the high-throughput data generations present challenges for many wet experimentalists to analyze and take full advantage of such rich and complex data. Here we present GeneCloudOmics, an easy-to-use web server for high-throughput gene expression analysis that extends the functionality of our previous ABioTrans with several new tools, including protein datasets analysis, and a web interface. GeneCloudOmics allows both microarray and RNA-Seq data analysis with a comprehensive range of data analytics tools in one package that no other current standalone software or web-based tool can do. In total, GeneCloudOmics provides the user access to 23 different data analytical and bioinformatics tasks including reads normalization, scatter plots, linear/non-linear correlations, PCA, clustering (hierarchical, k-means, t-SNE, SOM), differential expression analyses, pathway enrichments, evolutionary analyses, pathological analyses, and protein-protein interaction (PPI) identifications. Furthermore, GeneCloudOmics allows the direct import of gene expression data from the NCBI Gene Expression Omnibus database. The user can perform all tasks rapidly through an intuitive graphical user interface that overcomes the hassle of coding, installing tools/packages/libraries and dealing with operating systems compatibility and version issues, complications that make data analysis tasks challenging for biologists. Thus, GeneCloudOmics is a one-stop open-source tool for gene expression data analysis and visualization. It is freely available at http://combio-sifbi.org/GeneCloudOmics.

## Introduction

Multi-dimensional biological data is rapidly accumulating, and it is expected that the size of the data will exceed astronomical levels by 2025 (Stephens et al., 2015). This resulted in the development of computational tools that became vital in driving scientific discovery in recent times (Markowetz, 2017). A parallel increase in the development of online servers and databases has also been witnessed (Helmy et al., 2016), raising a new set of challenges related to the usability and maintenance of all these tools (Mangul et al., 2019). About half of the computational biology tools were found to be difficult to install, 28% of them are unavailable online in the provided URLs, and many others are missing adequate documentation and manuals (Mangul et al., 2019). The problem gets more complex with the limited computational and coding skills of two-thirds of the biologists who use these tools (Schultheiss, 2011). On the other hand, it was also noted that bioinformatics tools that are easy to install and use are highly cited, indicating wider usability by the community and a larger contribution to scientific discovery (Mangul et al., 2019). Thus, more web-based tools that avoid installation difficulties and operating system compatibility issues, simple point-and-click tools are required to tackle multi-dimensional omics datasets.

Gene expression profiling is widely used in biomedical research. They enable the investigation of expressed genes and their relevant pathways and cellular processes in a given time point or condition (Stark et al., 2019). Gene expression profiling is usually performed using RNA-Seq or microarray data since they detect the presence and quantify an RNA, the output indicator of an activated or deactivated gene (Yang et al., 2020). It also provides a deeper understanding of the biological system dynamics, growth or developmental process, drug effects or disease mechanisms through the differential gene expression (DGE) analysis (Piras et al., 2014, 2019; Simeoni et al., 2015; Hodgson et al., 2019; Bui et al., 2020; Wang et al., 2020). The DGE analysis determines genes with different expression levels between two or more conditions and statistically confirmed as differentially expressed (Pertea et al., 2016; Bui et al., 2020).

The analysis of gene expressions or transcriptomics data faces several challenges related to data size, quality, statistical analysis, visualization and interpretation of the results using current bioinformatics approaches (Mantione et al., 2014; Zou et al., 2019). Several bioinformatics or data science tools are available for addressing each of these challenges in the form of stand-alone software tools, web-server or R packages/Python libraries (Russo and Angelini, 2014; Poplawski et al., 2016; Velmeshev et al., 2016; McDermaid et al., 2019; Zou et al., 2019) (Table 1). However, most of the tools only provide a subset of analytics and require some level of programming skills. Often, the users need to move from one tool to another and this could lead to data compatibility issues (Chowdhury et al., 2019).

**TABLE 1:** Comparison between 30 different gene expression analysis tools.

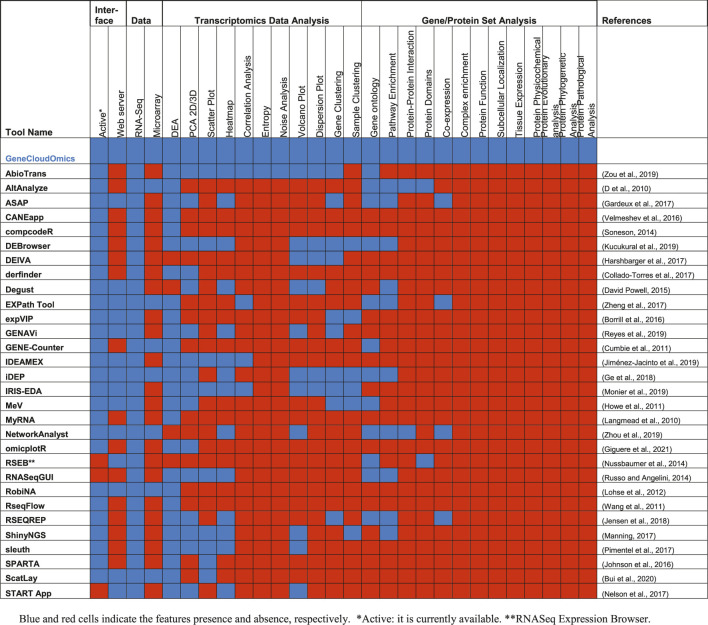

The analysis of gene expression data remains a burden for many biologists due to its intensive requirement of computational, statistical and programming skills that are lacking in two-thirds of biologists who use online biological resources (Schultheiss, 2011). Moreover, as mentioned above, most of the tools are individually scattered. Thus, there is a need to put the tools together in an easy-to-use manner with an intuitive GUI that will allow users to perform bioinformatics analyses with minimum computational skills and resources. In other words, a one-stop online server for transcriptomic data analysis that performs all essential steps of data import, pre-processing, statistical analyses, DGE identifications and functional interpretations of the results, through a friendly and simple user interface, is much needed.

Previously, we had developed ABioTrans as a stand-alone biostatistical tool for transcriptomics data analysis, including data pre-processing, statistical analyses, DGE and gene ontology (GO) classification (Zou et al., 2019). It is a downloadable executable that runs on any web browser with an interactive GUI (Table 2). However, as it is a stand-alone application written in R, the user needs to download it, install R or RStudio then run an installation script that installs all the required and up-to-date packages and dependencies. This was found to be challenging for some users as it requires a minimum level of programming familiarity, and several packages became incompatible with the new release of R (v4.0.0) in spring 2020. This is one common problem for most bioinformatics tools (Mangul et al., 2019). ABioTrans also needs approx. 10 min to download all packages before running. Hence, to provide users with a quick, ready-to-use that does not require regular system updates, a web server version is imminent.

**TABLE 2:** Features comparison between ABioTrans and GeneCloudOmics.

Architecture	ABioTrans	GeneCloudOmics
Application Type	Stand alone	Web-based
Requirements	R, RStudio, Web browser	Web browser
**Gene expression data**		
Supported transcriptome data	RNA-seq	RNA-Seq, Microarray
Supported transcriptome data formats	Gene expression table	Gene expression table microarray cel files
**Preprocessing and normalization**		
Low count filtering	Yes	Yes
Sequencing depth correction methods	TPM, RPKM, FPKM	TPM, RPKM, FPKM
Batch effect correction	UQ, RUV, TMM	UQ, RUV, TMM
**Biostatistics and Analytics**		
Dimension reduction	PCA	PCA Sparse PCA Self-organizing map (SOM)
Distribution fitting	Yes	Yes
Scatter plot	Yes	Yes
Correlation analysis	Pearson, spearman	Pearson, spearman
Entropy	Yes	Yes
Noise	Yes	Yes
**Differential expression analysis**		
Supporting plots	volcano plot, dispersion plot	volcano plot, dispersion plot
**Gene-based Clustering**		
Clustering algorithm	K-means clustering hierarchical clustering	K-means clustering hierarchical clustering
Visualization	Gene expression heatmap	Gene expression heatmap
**Sample-based Clustering**		
Clustering algorithm	K-means clustering on PC space	K-means clustering on PC space Random Forest clustering Self-organizing map (SOM)
Visualization	RF plot	RF plot, property plot, count plot, codes plot, distance plot and cluster plot
**Gene set analysis**		
Gene ontology	Yes	Yes
Gene ontology data	Local databases (NIH)	Online databases (UniProt)
Gene ontology visualization	Pie chart hairball graph	Bar chart
		Table
Pathway enrichment analysis	No	g:Profiler, Cytoscape (V)
**Protein set analysis**		
Protein-protein interaction	No	UniProt, Cytoscape (V)
Complex enrichment	No	CORUM
Protein function	No	UniProt
Subcellular localization	No	UniProt
Protein domains	No	UniProt
Tissue Expression	No	UniProt
Co-expression	No	GeneMANIA
Protein sequences	No	UniProt
Protein physicochemical analysis	No	Charge, GRAVY
Protein phylogenetic analysis	No	MAS, PGT., Chrom. Loc., G.Tree
Protein pathological analysis	No	UniProt

**FIGURE 1:**
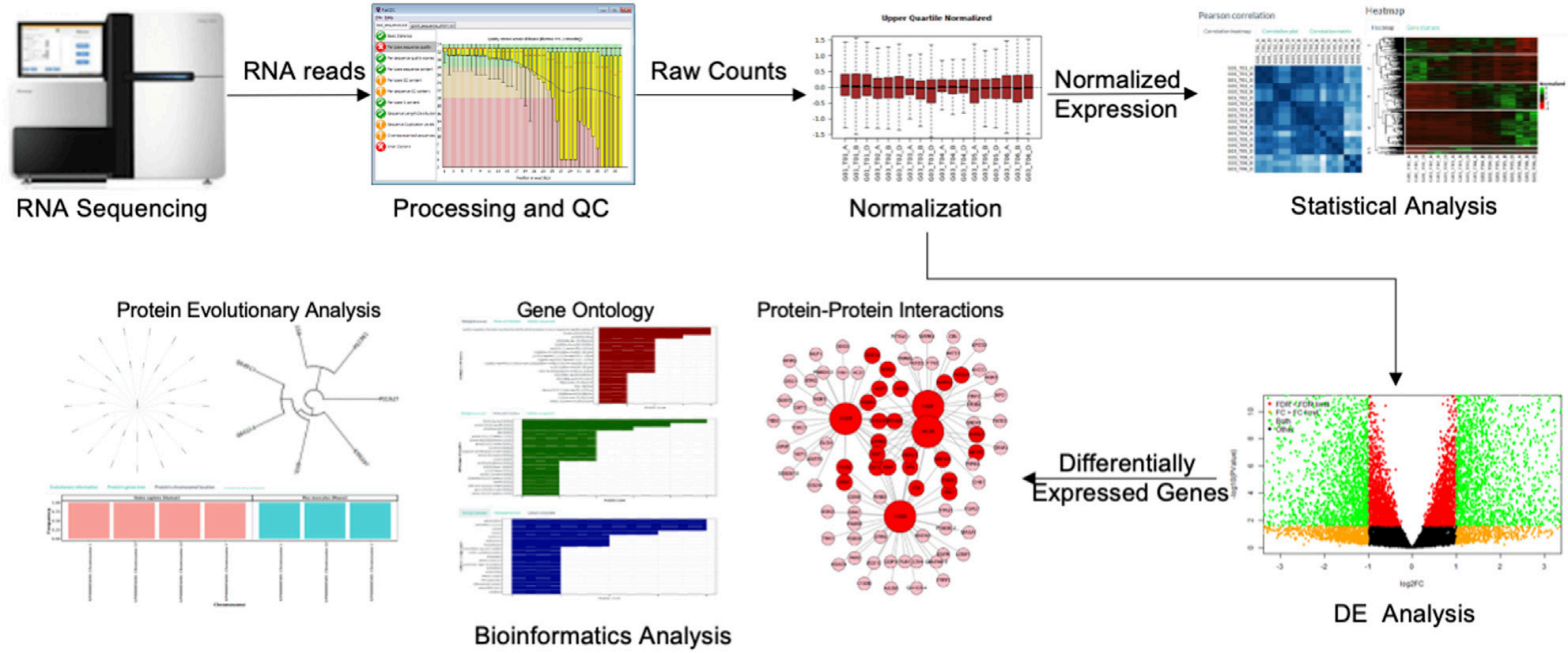
The gene expression profiling workflow. The RNA sequencer produces raw RNA read counts that are aligned on the cell’s genome and processed through the quality control (QC) steps. The raw read counts result from QC are next normalized and analyzed statistically to infer the differential gene expressions (DGEs) or other analyses such as Shannon Entropy, Correlations or PCA. Several bioinformatics analyses can also be performed on the list of DEG for functional inference.

To overcome the above-mentioned challenges, here we rebuilt ABioTrans as a new webserver and expanded its functionality to include several new analysis tools such as SOM, t-SNE, random forest clustering, and added further tools for bioinformatics functional analysis of gene and protein sets that includes PPI, protein complex analysis, evolutionary analysis, pathological analysis, physicochemical analysis, and more. We named this new revamped tool GeneCloudOmics, a web server for transcriptomics data analysis and gene/protein bioinformatics that is equipped with publication-ready plotting capabilities. GeneCloudOmics allows 12 biostatistical and data analytics tests and 11 bioinformatics tools for gene/protein datasets analysis and annotation (see Methods and Program Description). In addition, it provides direct data import from NCBI’s GEO databases through GEO accession numbers. GeneCloudOmics webserver, thus, relieves the burdens of installation and version compatibilities and is designed to be a quick one-stop transcriptomics (RNASeq and microarray) data analysis tool that provides the user with all the required steps for their analysis (Figure 1). Overall, the web server targets users without any computational or programming skills and provides them with a wide spectrum of hassle-free analytic tools.

## Methods and Program Description

### The Gene Expression Profiling Workflow

The gene expression analysis aims to identify genes expressed under a particular condition, treatment, developmental stage, or disease. This requires assessing thousands of gene expressions of multiple conditions in raw format, pre-processing and normalizing the expression levels, statistically analysing the data, identify DGEs between conditions and perform a functional analysis to elucidate the pathways and cellular functions of the DGEs (McDermaid et al., 2019) (Figure 1). GeneCloudOmics performs this workflow easily and smoothly on a web server as will be described below.

### Overview of GeneCloudOmics Web Server

**FIGURE 2:**
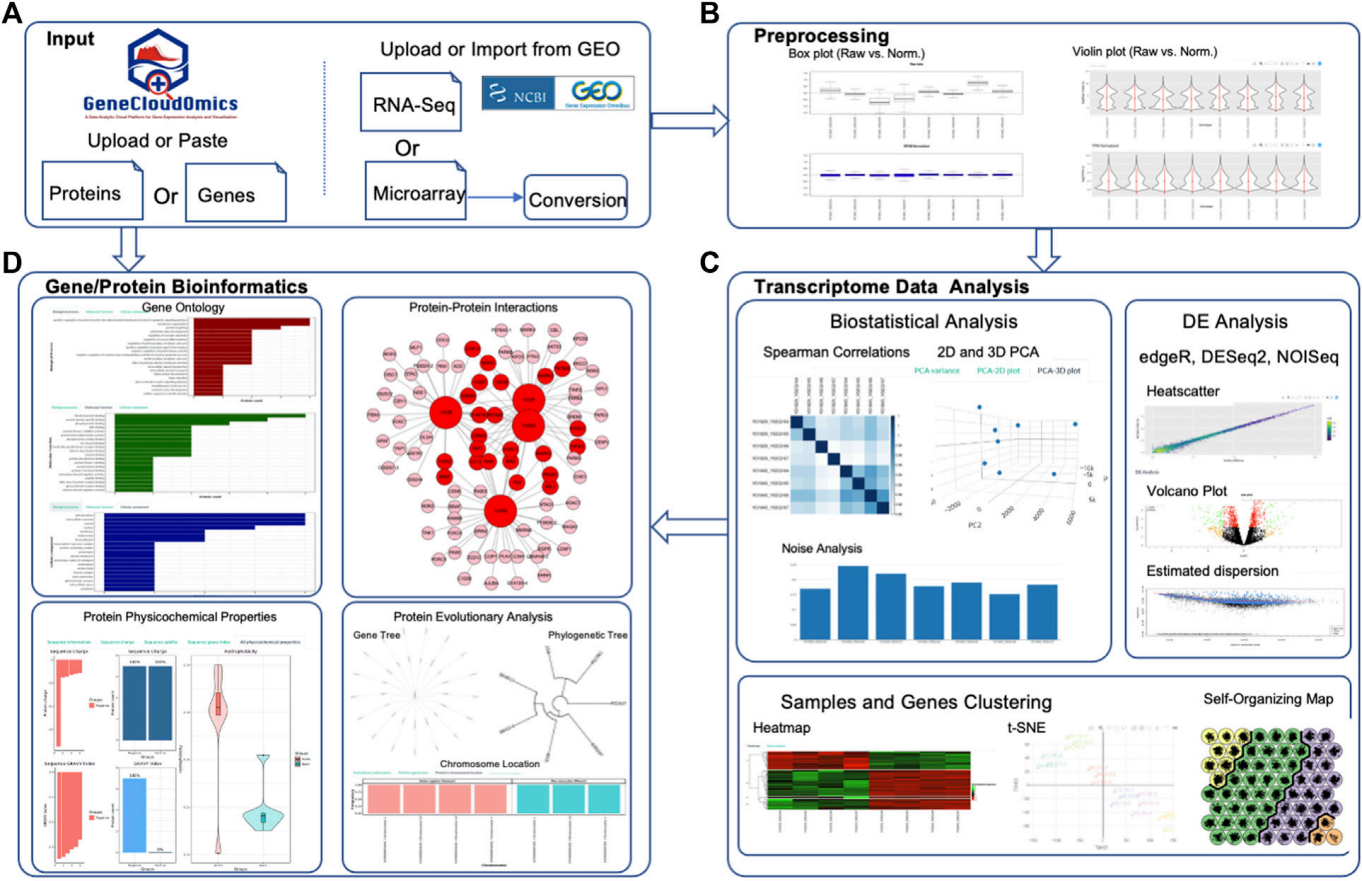
Schematic Overview of GeneCloudOmics. **(A)** RNASeq and Microarray data uploading, **(B)** Data Pre-processing, **(C)** Transcriptome Data Analysis (e.g. Correlation, DGE analysis and heatmap clustering), and **(D)** Gene or Protein Bioinformatics Analysis.

GeneCloudOmics provides users with a complete pipeline for analysing and interpreting their transcriptome data (Figure 2 and Table 2):

1) Data types: users input microarray (.cel files) or RNA-Seq data (raw or normalized read count table in. csv format). In addition, users can provide NCBI GEO database accession and GeneCloudOmics automatically imports the data from the database.
2) Pre-processing raw data using four different normalization techniques (RPKM, FPKM, TPM, RUV), then plotting the normalized data versus the raw data inbox and/or with violin plots. The pre-processed data can be downloaded into a CSV file.
3) Analyse the pre-processed data using nine different statistical tests (read normalization, scatter plots, linear/non-linear correlations, PCA, hierarchical clustering, k-means clustering, t-SNE clustering and SOM clustering) then plot the results of each test in a publication-ready quality.
4) Perform DGE analysis using three of the most commonly used methods DESeq2 (Love et al., 2014), NOISeq (Tarazona et al., 2015) and EdgeR (Robinson et al., 2009) with a single interface for choosing the parameters for each of the methods and in a similar way to plot the results in volcano or dispersion plots. The user can then download the results as a CSV file and the plots as or PNG or PDF.
5) Functionally interpret the DGEs or proteins set using 11 different bioinformatics tools (listed in detail below and in Table 2) that help the user perform essential enrichments and annotations to the gene/protein sets such as pathway enrichment analysis, gene ontology (GO) enrichment, PPI, and protein function enrichment. All the tests are performed through the same interface which allows the user to upload or paste a list of genes or proteins, choose the test parameters, run the analysis, and plot the results, using the standard visualization provided, or download them. The gene/protein set interpretation features are independent from the DGE analysis and can be used separately with any gene/protein set as a stand-alone feature (see demonstration sections below).
6) Creating an analysis report that summarizes and gathers all analyses of the user. In each test or analysis, the user can choose “Add to Report” option which will add the plot and the analysis title to the analysis report. When the user clicks “Analysis report” link in the main menu, the system generates an HTML report containing all the selected plots. The user can then download the report as a PDF.

### Data Analytics Features

GeneCloudOmics accepts both gene expression matrix from RNA-Seq and raw microarray CEL file formats, either through data upload forms or via direct import from GEO database. Examples of valid input files are hyperlinked at each upload section to aid the user with the input files.

For RNA-Seq, two input files are required: 1) gene expression matrix, and 2) metadata table. The gene expression matrix should contain estimated abundance (either raw count or normalized) of all genes for all samples in the experiment; and the metadata table should specify experimental conditions (e.g., Control, Treated, etc.) for each sample listed in the expression matrix. Depending on target analysis, the user can upload supporting files including gene length and list of negative control genes to facilitate the pre-processing step.

For microarray, the user can upload CEL files to GeneCloudOmics, upon which matrix of gene expression level will be extracted and the user can proceed to subsequent analyses. The data obtained directly from GEO database will undergo an initial exploratory analysis that overviews the quality of data using several plots.

Next, the transcriptomics data is processed and analyzed using the following analytics:

1- Data preprocessing: Preprocessing includes two steps: 1) low-expression gene filtering, and 2) data normalization. Removal of lowly expressed genes is crucial to reduce the effects of measurement noise, and consequently improve the number of differentially expressed genes (Sha et al., 2015). GeneCloudOmics provides the option for the user to indicate the minimum expression value and the minimum number of samples that are required to exceed the threshold for each gene. If input data contain raw read counts, the user can choose one of the normalization options: Fragments Per Kilobase Million (FPKM), Reads Per Kilobase Million (RPKM), Transcripts Per Kilobase Million (TPM) (Li et al., 2015), Remove Unwanted Variation (RUV) (Risso et al., 2014) or Upper Quartile (Bullard et al., 2010). FPKM, RPKM and TPM option perform normalization for sequencing depth and gene length, whereas RUV and upper quartile eliminate the unwanted variation between samples. To check for sample variation, Relative Log Expression (RLE) plots (Gandolfo and Speed, 2018) of input and processed data are displayed for comparison.
2- Transcriptome-wide distributions: Gene expressions are known to follow certain statistical distributions such as power-law or lognormal (Furusawa and Kaneko, 2003; Bengtsson et al., 2005; Beal, 2017), which has been applied to determine a suitable gene expression threshold for low signal-to-noise expression cut-off (Piras et al., 2014, 2019; Piras and Selvarajoo, 2015; Simeoni et al., 2015; Bui and Selvarajoo, 2020). GeneCloudOmics can compare the cumulative distribution function (CDF) of transcriptome-wide expression with six model distributions: Log-normal, Log-logistic, Pareto (or power law), Burr, Weibull, and Gamma. The goodness-of-fit for each distribution is measured by the Akaike information criterion (AIC), from which the user can choose the best-fitted distribution and select threshold for low-expression gene removal.
3- Scatter plot: Scatter plot compares any two samples (or two replicates) by displaying the respective expression of all genes in 2D space. As gene expression data is densely distributed in the low-expression region, making the scatter dots indistinguishable, GeneCloudOmics also overlays the estimated 2D kernel density on the scatter to better visualize the scatter dot density. The scatter plot also shows how variable the gene expressions are between any two samples. The wider the scatter, the less similar the global responses and vice-versa (Piras et al., 2014).
4- Pearson and Spearman correlations: GeneCloudOmics can evaluate the transcriptome-wide relationship between any two samples by linear (Pearson) and monotonic non-linear (Spearman) correlations, displayed in 1) actual values in a table or 2) as a heat map.
5- Principal components analysis and sample clustering: Principal Components Analysis (PCA) is used for simplifying the high-dimensional gene expression data into two or more dimensions, termed the principal components. Doing so, the whole transcriptome data can be visualized on a 2D or 3D plot. Each principal component is a linear combination of the original variables, hence, we can ascribe meaning to what the components represent. From the principal components, GeneCloudOmics can cluster the samples into groups based on their similarity by *K*-means clustering.
6- t-distributed stochastic neighbour embedding (t-SNE): t-SNE is another dimensionality-reduction approach that reduces the complexity of transcriptomic data (Cieslak et al., 2020). GeneCloudOmics introduces an intuitive interface that allows performing t-SNE analysis on the processed untransformed transcriptomic. The user can also choose to log transform the data before submission. Sample clustering by *K*-means is also applied on the t-SNE transformed dataset upon user selection.
7- Shannon entropy: GeneCloudOmics adopts the formula of Shannon entropy (Shannon, 1948) from information theory to measure the disorder of a high-dimensional gene expression sample, where a higher value indicates higher disorder. As the original formula for entropy is restricted to discrete variables, GeneCloudOmics has to discretize gene expression data (which is a continuous variable) by histogram-based binning; the number of bins are determined by Doane’s rule (Doane, 1976; Piras et al., 2014).
8- Averaged transcriptome-wide noise: Averaged transcriptome-wide noise quantifies the variability between gene expression scatters of all replicates in one experimental condition (Piras et al., 2014). The noise is defined as the average of variance (σ^2^) of expression divided by the square mean expression (μ^2^), for all genes between all possible pairs of replicates (Piras et al., 2014).
9- Differential Expression (DE) Analysis: DE analysis identifies genes that are statistically different in expression levels between any two selected conditions. GeneCloudOmics implements three popular DE methods: edgeR, DESeq2 and NOISeq. In case there are no replicates available for any of the experimental condition, technical replicates can be simulated by NOISeq. To better visualize differentially expressed genes among the others, a volcano plot (plot of log_10_-p-value and log_2_-fold change for all genes) distinguishing the DE and non-DE genes is displayed. Plot of dispersion estimation, which correlates to gene variation, is also available in DESeq2 and EdgeR method.
10-Heatmap and gene clustering: This function clusters differentially expressed genes (result from previous step) into groups of co-varying genes. Expression levels of DE genes first undergo scaling defined by $z_{j}\left(p_{i}\right)=\left(x_{j}\left(p_{i}\right)-\bar{x_{j}}\right)/\sigma_{x_{j}}\;$ where $z_{j}\left(p_{i}\right)$ is the scaled expression of the *j* ^*th*^ gene, $x_{j}\left(p_{i}\right)$ is an expression of the *j* ^*th*^ gene in sample *p*_*i*_, $\bar{x_{j}}$ is the mean expression across all samples and $\sigma_{x_{j}}$ is the standard deviation (Simeoni et al., 2015). Subsequently, Ward hierarchical clustering is applied on the scaled expression.
11-Random forest-based clustering: GeneCloudOmics uses RAFSIL (Pouyan and Kostka, 2018), which is a random forest based similarities learning method between single cells from RNA sequencing experiments. RAFSIL utilizes random forest algorithm to learn the pairwise dissimilarity among cells/samples, which in turn is used as an input to the K-means clustering algorithm. The resultant data is subsequently enhanced using t-SNE-reduced dimensions, to reveal clearer clusters of cells/samples.
12-Self-Organizing Map (SOM): SOM is a dimensionality reduction technique that produces a two-dimensional, discretized representation of the high-dimensional gene expression matrix (Yin, 2008). GeneCloudOmics provides a SOM function that outputs five different plots: property plot, count plot, codes plot, distance plot and cluster plot.

### Bioinformatics Tools

DGE analysis usually outputs a list of genes that are statistically determined as differentially expressed genes (DEGs). Next, the list of DEGs is analyzed, interpreted, and annotated to learn more about the functions, pathways, and cellular processes where these genes are involved, for example, diseases they are associated with or perform other investigations on the properties of those genes/proteins (such as phylogenetic or physiochemical analyses). Most of the currently available DGE analysis tools do not include bioinformatics features for gene set analysis or include only a few basic analyses such as GO and pathways enrichment (Table 1). Even our previous tool, ABioTrans, only provides one GO tool for interpreting the DEGs. In GeneCloudOmics, we redesigned the GO feature to be dynamic by reading the GO terms associated with the genes/proteins directly from UniProt Knowledgebase (Bateman, 2019) then visualize each of the three GO domains (cellular component, molecular function and biological process) in independent tabs. Furthermore, we have introduced 11 new bioinformatics tools that can be performed on a given gene/protein dataset.

**FIGURE 3:**
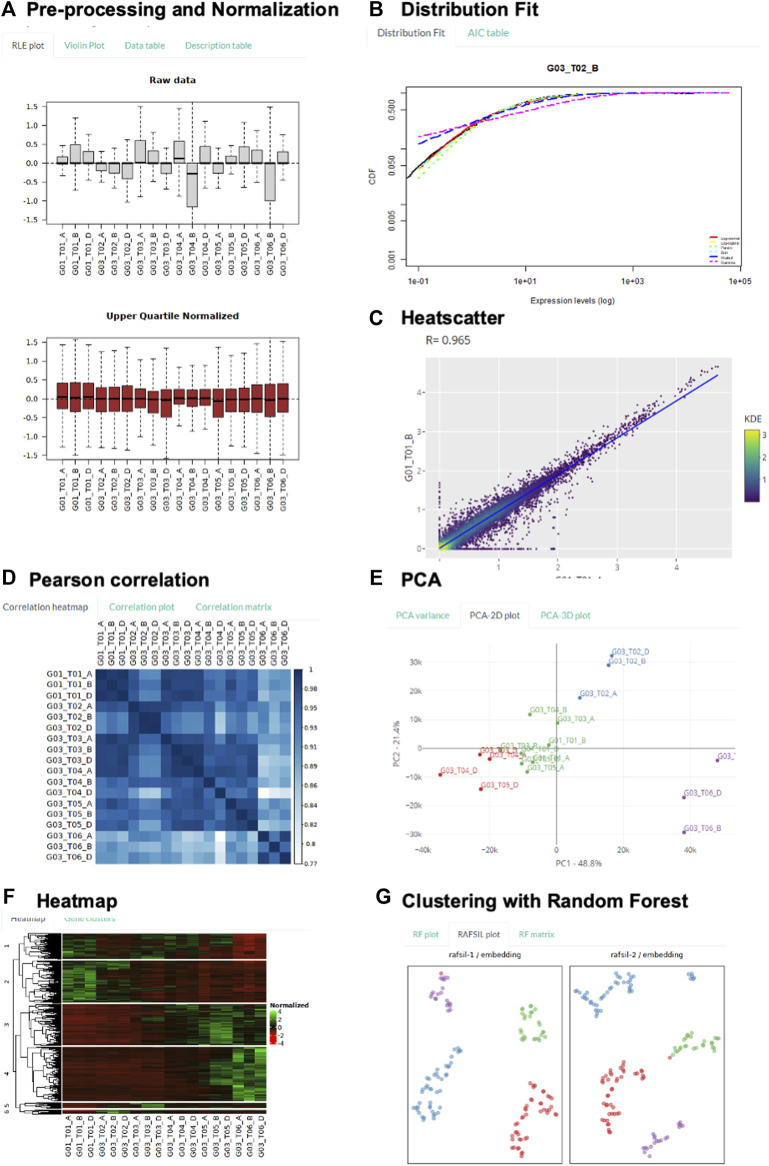
Demonstration of Key Transcriptomic Analysis using GeneCloudOmics. **(A–F)** using bulk-RNASeq human T-regulatory cell differentiation data, and **(G)** using single-cell RNASeq mouse distal lung epithelium data. **(A)**: RLE plot of raw and normalized data, showing sample variation reduced after normalization. **(B)**: Comparing transcriptome-wide distribution with six model distributions to select suitable expression cut-off threshold. **(C)**: Between-replicate transcriptome-wide variation visualized by scatter plot. **(D)**: Pairwise Pearson correlation between all samples. **(E)**: Principal component analysis visualizes all sample data points in 2 dimensions. **(F)**: Hierarchical clustering reveals common expression patterns throughout the T cell differentiation process, visualized by heat map of expression level. **(G)**: Random Forest clustering divides single cells according to their developmental stages.

1) Pathways Enrichment Analysis: For a given gene or protein set, GeneCloudOmics uses g:Profiler (Raudvere et al., 2019) to perform a pathway enrichment analysis and displays the results as a network where the nodes are the pathways and the edges are the overlap between the pathways (Figure 3A). We use Cytoscape. JS for the network visualization (Franz et al., 2015) and through this, the network properties such as colour and layout can be changed and the final network can be downloaded.
2) Protein-Protein Interaction: GeneCloudOmics provides the user with an interface where they can upload a set of proteins (UniProt accessions) and get all the interactions associated with them. The interactions are visualized as a network where the nodes are the proteins, and the edges are the interactions, and the node size corresponds to the number of interactors of the protein. This feature uses Cytoscape. JS for the network visualization (Franz et al., 2015).
3) Complex Enrichment: The identification of the subunits of the protein complexes is important to understand the protein functions and the formation of these macromolecular machines. GeneCloudOmics provides the user with a complex enrichment feature that allows identification of proteins in the provided dataset that are part of a known protein complex using CORUM databases (Giurgiu et al., 2019).
4) Protein Function: UniProt provides a detailed function for thousands of protein sequences. The protein function feature retrieves protein function information from UniProt of a given protein set.
5) Protein Subcellular Localization: Protein localization critically affects a protein function. The protein subcellular localization feature provides the user with an interface to UniProt to get the subcellular localization information for a given list of proteins.
6) Protein Domains: The protein domains are functional subunits of the proteins that contribute to their overall function. GeneCloudOmics provides the user with a protein domain feature that retrieves the domain information from UniProt for a given list of proteins.
7) Tissue Expression: The distinct expression profile of genes and proteins per tissue is what gives different tissues the suitability for their functions. The tissue expression feature in GeneCloudOmics provides the user with tissue expression information from UniProt for each protein in a given list.
8) Gene Co-expression: The co-expression analysis is a common analysis that assesses the expression level of different genes to identify simultaneously expressed genes, which indicates that they are controlled by the same transcriptional mechanism (Vella et al., 2017). GeneCloudOmics provides the user with an interface where they can submit a co-expression query to GeneMANIA (Franz et al., 2018).
9) Protein Physicochemical Properties: For a given set of proteins (UniProt accessions), this feature provides the user with complete sequences of them in a single FASTA file and allows the user to investigate their physicochemical properties, sequence charge, GRAVY index (Kyte and Doolittle, 1982) and hydrophobicity. The full sequences of the proteins are automatically obtained from UniProt Knowledgebase while the physicochemical properties are investigated and plotted using the UniProtR package (Bateman, 2019; Soudy et al., 2020)
10) Protein Evolutionary Analysis: For a given set of proteins, this feature provides the user with a phylogenetic and evolutionary analysis that includes multiple sequence alignment (MSA) of the protein sequences, clustering based on the amino acid sequences, chromosomal locations, or gene trees.
11) Protein Pathological Analysis: Several diseases are associated with the malfunction of certain genes or proteins. The disease-protein association is collected in different online resources such as OMIM databases (Amberger et al., 2019), DisProt (Hatos et al., 2020) and DisGeNET (Piñero et al., 2020). GeneCloudOmics provides the user with an interface that retrieves the disease-protein association from online databases for a given list of proteins and visualizes disease-protein association as a bubble.

The features that communicate with UniProt use UniProtR, an R package for data retrieval and visualization from UniProt (Soudy et al., 2020). Since all the bioinformatics features only accept gene names (gene symbol) or UniProt accessions, we provide the user on each page with links to two ID converters UniProt ID mapping (Bateman, 2019) and g:Convert (Raudvere et al., 2019) to convert their identifiers to gene names or UniProt accessions. All the analyses are either performed on the uploaded data or involve connecting to a remote server such as UniProt Knowledgebase. GeneCloudOmics does not store any uploaded data and does not contain any databases.

## Demonstration of Genecloudomics Utility

### Transcriptome Analysis Features

We performed a demonstration of transcriptomic analysis with a recent study on the time-resolved bulk cell RNA-Seq profile of human T regulatory cell differentiation (Schmidt et al., 2018). In the study, human T regulatory cells were isolated from peripheral blood; upon which differentiation was induced by adding TGF-ß factor, in comparison to naïve (unstimulated) T regulatory cells as the control group. At the indicated time points (0, 2, 6, 24, 48 h, 6 days), the cells were collected for RNA extraction and sequencing. Here, we illustrate how GeneCloudOmics was used for data pre-processing (normalization and filter low count), differential analysis, and data clustering.

Firstly, unwanted variation among samples was removed by Upper Quartile normalization. The RLE plot clearly illustrates the normalization effects: high between-sample variation in raw data versus low variation after normalizing (Figure 3A). We also utilized the transcriptome-wide distribution fitting feature to determine the expression threshold for low count filtering (Figure 3B) (Simeoni et al., 2015; Bui et al., 2020). The threshold of five counts was selected because from this expression level onwards, transcriptome-wide expression was observed to follow most of the model statistical distributions.

Next, pairwise scatter, pair-wise sample correlation, and PCA were used to visualize the global relationship of all data samples, through which initial assessment on data quality can be gauged. For example, the low between-replicate variation in contrast with high between-condition variation could be shown by the width of scatter plots (Figure 3C, Supplementary Figure S1A). It is further illustrated by the correlation heatmap, in which the replicates of the same condition all show close-to-unity Pearson correlation value along the diagonal axis (Figure 3D); whereas decreasing correlation value with time was observed along the edge of the heatmap. This information is of high importance because low correlation or high variance across replicates will negatively impact the power to detect differentially expressed genes. Clustering of replicates of similar time points was further illustrated in PCA and t-SNE plots, in which the last time point (T06 - when the T cells were fully differentiated) formed a distinct cluster from the transitioning time points (Figure 3E, Supplementary Figure S1B). From these analyses, we knew that the data show low variation between replicates and that gene expression globally changed along the differentiation time.

We performed differential expression (DE) analysis with all three supported DE methods: EdgeR, DESeq2 and NOISeq; and presented the analysis conducted with DESeq2 (Supplementary Figures S1D,E). The last time point (T06) was compared against the control group (T01) to extract DE genes in the differentiation process (with 0.05 *p-*value and 2-fold expression threshold). Two important steps in DESeq 2 were visualized: 1) the estimation of gene-wise dispersion and empirical shrinkage of these estimates to produce a more accurate dispersion estimate for actual gene count modelling (Supplementary Figure S1E); and 2) the volcano plot that summarizes DESeq2 p-value and expression fold difference for every gene (Supplementary Figure S1E). The list of all 5,033 differentially expressed genes (3,017 up, 2,016 down) was also listed in a separate table. Finally, the DE genes were channelled into heatmap gene clustering feature, from which DE genes sharing similar patterns of gene expression change throughout the differentiation process were identified (Figure 3F). Four common expression patterns were observed: 1) gradual decrease (Group 2), 2) gradual increase (Group 3 and 4), 3) initial increase followed by decrease (Group 5 and 6), and 4) sharp decrease, followed by a gradual increase, and finally decrease (Group 1).

To further illustrate the dimension-reduced visualization features t-SNE and random forest clustering, we used another single-cell RNA-Seq dataset of distal lung epithelium (Treutlein et al., 2014). The study measured gene expression of a total 198 individual mouse lung epithelial cells at four different stages (E14.5, E16.5, E18.5, adult) throughout development. Sample clustering by k-means on t-SNE1 and t-SNE2 space divided the cells into clusters that are aligned with their respective development stages (Supplementary Figure S1C and Additional File S1): Cluster 1 contains mostly E18 cells, Cluster 2 and 3 contain mostly AT2 cells, Cluster four contains mostly E16 cells, and Cluster 5 contains mostly E14 cells. Finally, clustering by random forest approach (Pouyan and Kostka, 2018) determined the number of clusters as the number of types of cells provided in the input metadata table and subsequently grouped the cells according to their developmental stages (Figure 3G).

### New Bioinformatics Features

To demonstrate the utility of the bioinformatics section, we used data from a differential proteomics analysis that was conducted using the AGS cell lines of gastric cancer (GC) (Saralamma et al., 2020). The AGS cells were treated with Scutellarein, a flavone known for its anticancer effect. The study identified 41 proteins that are differentially expressed in AGS when treated with Scutellarein, 24 of them were downregulated and 17 were upregulated.

**FIGURE 4:**
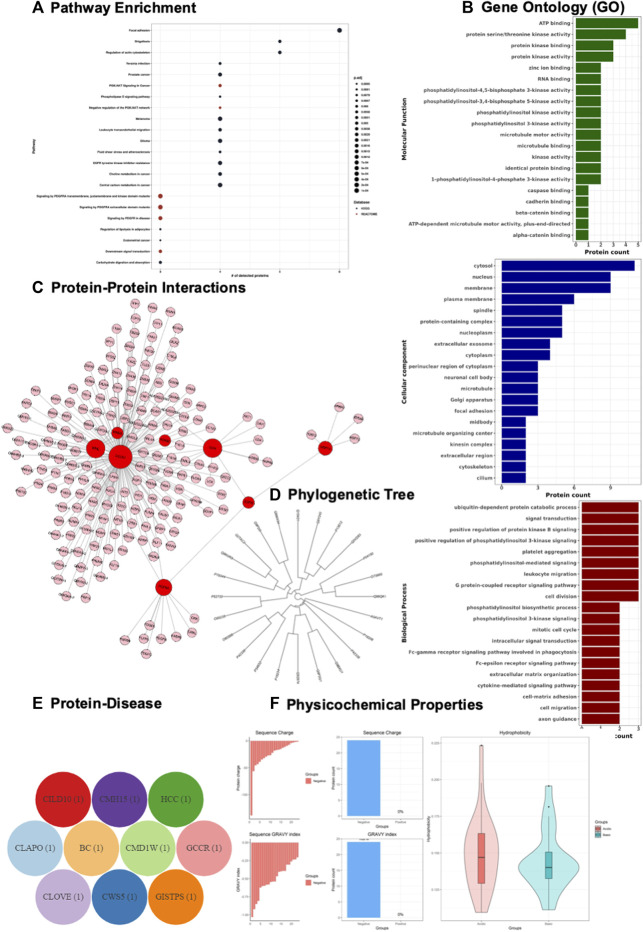
Demonstration of Gene and Protein Bioinformatics Analysis using GeneCloudOmics. **(A)** pathway enrichment analysis, **(B)** gene ontology (GO), **(C)** protein-protein interaction, and **(D)** protein phylogenetic analysis, **(E)** protein pathological analysis, and **(F)** Protein physicochemical properties (acidity, charge and hydrophobicity) of Scutellarein treated AGS cell lines of gastric cancer proteomics dataset.

Pathway analysis shows that the down-regulated proteins are associated with movement of cellular or subcellular components and platelet activation (Figure 4A), while that pathways enrichment for the up-regulated proteins did not result in any significantly enriched pathways. Functional analysis is retrieved, visualized, and represented as Gene Ontology (GO) terms (Biological process; Molecular function; Cellular component). The down-regulated profile shows cell processing components including cell cycle, cell division, and cell migration (Figure 4B), while the up-regulated profile shows a regulation of apoptotic process including positive and negative regulation associated with cytokine-mediated signalling pathway (Supplementary Figure S2A). Protein-protein interaction (PPI) network of both down-regulated and up-regulated proteins retrieved from UniProt (Bateman, 2019) and visualized using Cytoscape. JS (Franz et al., 2015) and GeneCloudOmics protein interaction feature (Figure 4C, Supplementary Figure S2B).

GeneCloudOmics internally uses ClustalOmega (Sievers and Higgins, 2021) to perform a multiple sequence alignment (MSA) which was used to investigate and visualise the homogeneity among protein sequences (Figure 4D and Supplementary Figure S2C). Pathological analysis of the protein list is a crucial step in data interpretation for connecting computational output with biological data, so the protein accession list is mapped to OMIM database disease IDs for providing information about diseases associated with proteins (Figures 4E and Supplementary Figure S2D). Physicochemical analysis of the two sets of proteins shows that sequence charge of 100% of the down-regulated proteins is negative while in the up-regulated proteins it is 94% negative and 6% positive (Figures 4F and Supplementary Figure S2E).

## Summary and Future Developments

In this paper, we have introduced a new webserver, GeneCloudOmics, for gene expression data analysis using a simple easy-to-use GUI that contains 23 data analytic and bioinformatics tools. This is the largest number of tools in any current webserver to our knowledge (Table 1). We have demonstrated the utility of key functions using recently published human T regulatory cell differentiation and mouse distal lung epithelium RNA-Seq dataset (Risso et al., 2014; Schmidt et al., 2018) and Scutellarein treated AGS cell lines of gastric cancer proteomics dataset (Saralamma et al., 2020).

In the next few years, GeneCloudOmics could be extended to support additional types of high throughput data, on top of RNA-Seq or microarrays. The plan includes supporting the analysis of proteomics, metabolomics, chromatin immunoprecipitation sequencing (ChIP-Seq) and cross-linking immunoprecipitation (CLIP-Seq) data. In addition, we hope to continue improving the transcriptome data analysis by adding new features such as other DGE methods [e.g. Limma (Dias-Audibert et al., 2020) and ScatLay (Bui et al., 2020)], sample overlap analysis (Venn diagram), additional data plots (e.g. density plot) and support for Gene Set Enrichment Analysis (GSEA) (Subramanian et al., 2005). The gene and protein IDs could also be extended to support different IDs, so the user is not restricted to use gene names and UniProt accessions only.

## Data Availability

The GeneCloudOmics web server can be freely accessed at http://combio-sifbi.org/GeneCloudOmics. The software is written using the open-source R programming language (R: a language and environment for statistical computing) and the Shiny framework (Web Application Framework for R [R package shiny version 1.6.0], 2021). A Docker container image is also available (docker pull jaktab/GeneCloudOmics-webserver:latest). GeneCloudOmics is optimized for Google Chrome. Details on the R packages used in GeneCloudOmics, their versions and sources are available in Supplementary Table S1 and in the tool documentation on GitHub (https://github.com/cbio-astar-tools/GeneCloudOmics).

## Supplementary Material

Supplementary Figure S1 Demonstration of GeneCloudOmics transcriptomic analysis features on **(A–E)**: bulk-RNASeq human T regulatory cell differentiation data, and **(C)**: single-cell RNASeq mouse distal lung epithelium data: **(A)**: Between-replicate and between-condition transcriptome wide variation visualized by scatter plot. **(B, C)**: t-SNE (right) visualize all sample data points in 2 dimensions for **(B)**: bulk-cell RNA-Seq human T cell data, and **(C)**: single-cell RNASeq distal lung epithelium data. **(D, E)**: Estimated dispersion **(D)** and resulting volcano plot **(E)** from DESeq2 differential expression analysis with p-value threshold at 0.05 and expression fold threshold at 2.

Supplementary Figure S2 Protein bioinformatics analysis for the upregulated proteins set. **(A)** gene ontology (GO), **(B)** protein-protein interaction, and **(C)** protein phylogenetic tree, **(D)** protein pathological analysis, and **(E)** Protein physicochemical properties (acidity, charge and hydrophobicity).

Supplementary Table S1 The list of the R packages used in GeneCloudOmics.
