# Supplementary figures and images for: GeneCloudOmics: A Data Analytic Cloud Platform for High-Throughput Gene Expression Analysis
Source: Front Bioinform. 2021 Nov 25;1:693836. doi: 10.3389/fbinf.2021.693836 (PMC9581002; doi:10.3389/fbinf.2021.693836)

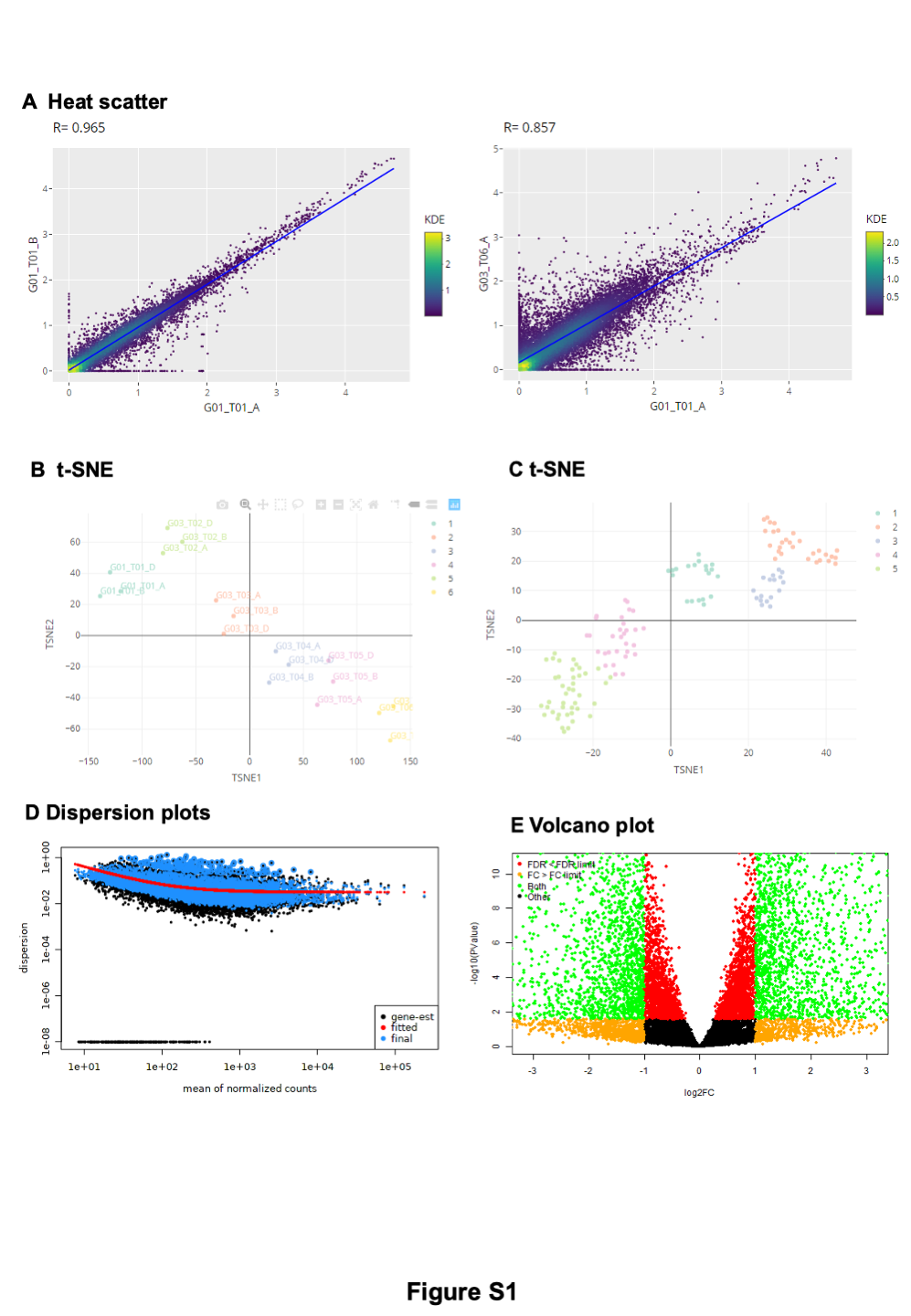

Supplement: Supplementary file 1 [file Image1.tiff]

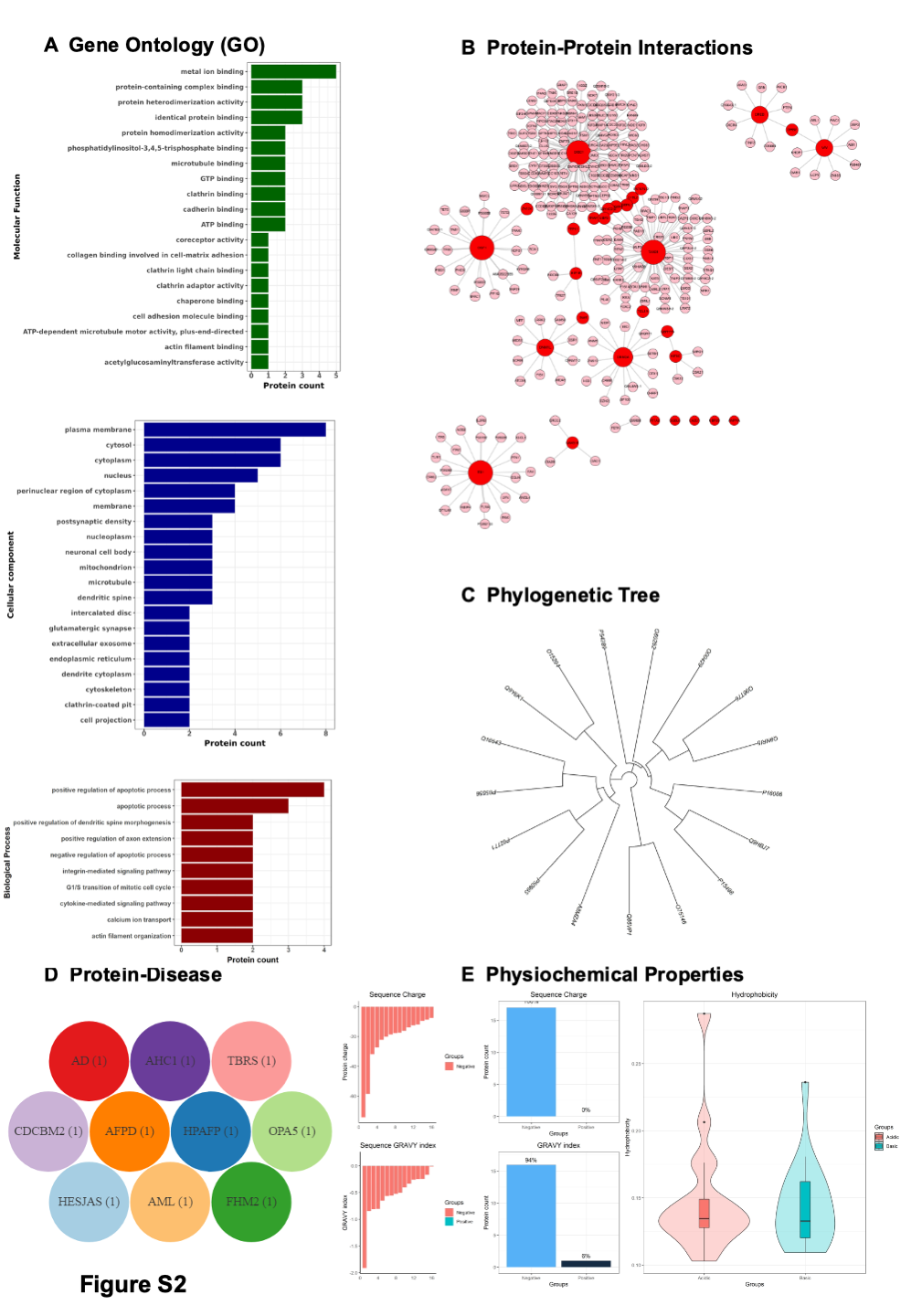

Supplement: Supplementary file 2 [file Image2.tiff]
